# The “Handling” of power in the physician-patient encounter: perceptions from experienced physicians

**DOI:** 10.1186/s12909-016-0634-0

**Published:** 2016-04-18

**Authors:** Laura Nimmon, Terese Stenfors-Hayes

**Affiliations:** Centre for Health Education Scholarship, Vancouver, Canada; Department of Occupational Science and Occupational Therapy, 429 – 2194 Health Sciences Mall, Faculty of Medicine, University of British Columbia, Vancouver, British Columbia V6T 1Z3 Canada; Department of Learning, Informatics, Management and Ethics, Karolinska Institutet, 17177 Stockholm, Sweden

**Keywords:** Physician-patient relationship, Patient-centred care, Qualitative research

## Abstract

**Background:**

Modern healthcare is burgeoning with patient centered rhetoric where physicians “share power” equally in their interactions with patients. However, how physicians actually conceptualize and manage their power when interacting with patients remains unexamined in the literature. This study explored how power is perceived and exerted in the physician-patient encounter from the perspective of experienced physicians. It is necessary to examine physicians’ awareness of power in the context of modern healthcare that espouses values of dialogic, egalitarian, patient centered care.

**Methods:**

Thirty physicians with a minimum five years’ experience practicing medicine in the disciplines of Internal Medicine, Surgery, Pediatrics, Psychiatry and Family Medicine were recruited. The authors analyzed semi-structured interview data using LeCompte and Schensul’s three stage process: Item analysis, Pattern analysis, and Structural analysis. Theoretical notions from Bourdieu’s social theory served as analytic tools for achieving an understanding of physicians’ perceptions of power in their interactions with patients.

**Results:**

The analysis of data highlighted a range of descriptions and interpretations of relational power. Physicians’ responses fell under three broad categories: (1) Perceptions of holding and managing power, (2) Perceptions of power as waning, and (3) Perceptions of power as non-existent or irrelevant.

**Conclusions:**

Although the “sharing of power” is an overarching goal of modern patient-centered healthcare, this study highlights how this concept does not fully capture the complex ways experienced physicians perceive, invoke, and redress power in the clinical encounter. Based on the insights, the authors suggest that physicians learn to enact ethical patient-centered therapeutic communication through reflective, effective, and professional use of power in clinical encounters.

## Background

Although the role power plays in medical interactions is becoming increasingly recognized as an important area of inquiry, [1–12] research is still nascent in this area. When power is depicted in the physician-patient encounter, it is rendered as something physicians own and yield to their own advantage with little conscious awareness [13, 14]. To our knowledge, there is no qualitative research that has explored with experienced physicians themselves how they both view power in the physician-patient encounter and the ways they are intentional with its use. Physicians’ qualitative descriptions of their awareness of power is important to capture in the context of modern healthcare eschewing a paternalistic approach to physician-patient relationships, and espousing values of dialogic, egalitarian, patient centered care [15]. These ideals are pervasive, for example, in depictions of reflective physicians who “share power” through engaging in shared decision making practices with patients [16–21]. With the current emphasis in the healthcare literature on the equal sharing of power as an integral element of patient centered therapeutic communication, [17] we aimed to explore how experienced physicians themselves perceive power and its distribution in the physician-patient encounter.

### Theoretical framework

#### Bourdieu’s social theory

Under a broader paradigm of sociocultural theory, we draw on Bourdieu’s [22] work which emphasized that whatever power language possesses is a power ascribed by the social institution (e.g. social institution of medicine) with which the speaker is associated. We also apply Bourdieu’s theoretical concept of “habitus” which is described as a set of learned “dispositions” and inclines individuals to act and react in certain ways. Through a myriad of processes, such as medical training and education, an individual acquires a set of “dispositions” that become second nature and embodied. These dispositions generate practices, perceptions, behaviour, and attitudes which reflect the social conditions (e.g. medical training) within which they were acquired.

Bourdieu’s concept of doxa further elaborates his notion of habitus. Doxa is a conceptual tool that can be used to make sense of how physicians subconsciously accept and internalize attitudes, knowledge, beliefs and values of the institutional and organizational culture of medicine without knowing they are doing so [23]. According to Bourdieu, doxa is transmitted through the body, language and dispositions towards things that are below a level consciousness [23]. This subconscious internalization of physicians’ position of power is evident, for example, in empirical research where physicians have described themselves as at the top of the healthcare hierarchy; as the “leaders” and “decision makers” who have “training”, “knowledge” and “skills” that are more valuable than that of other health professionals [11].

According to Bourdieu [22] when individuals interact they do so in specific social context: “the field”, which in turn shapes their practices, perceptions and attitudes. There are various levels of social fields in medicine; such as macro level field of medicine or healthcare, the median level field of the hospital, and the micro level field of patient-doctor interactions [24]. Habitus can thus change over time in relation to exposure to specific social fields, and is not static or permanent. Social fields are sites where positions of power are determined by the distribution of different kinds of capital, which can include, for example, “cultural capital” (e.g. medical knowledge & skills) or “symbolic capital” (e.g. accumulated professional prestige or honour). This “symbolic power” can be understood as an everyday form of power (rather than the power of physical force) and is deployed in social context. A social field is thus a site of negotiation in which individuals seek to maintain or alter the distribution of different forms of capital.

### The nature of power

“Power” as used in this study is defined as a relational co-constructed process and represents a potential to exert influence [25]. Power is present in all interpersonal relationships; there is thus no interaction in which power is not relevant in healthcare. Power is neither positive nor negative, but “comes into being” when it is put into action through “strategies” [26]. These strategies are observable in that they are expressed through language; language is tied to structures of power such as the social institution of medicine [22]. Physicians can exert power by drawing on the legitimized institutional language of medicine they are affiliated with by virtue of their qualifications and training [22].

A challenge facing physicians is that at a micro level of interaction, the very nature of their relationship with patients is asymmetrical. This unequal relationship is a product of physicians possessing legitimized, referent, and expert power [22, 27] and patients being reliant on physicians to provide the care and services they need [28]. Although a caring, respectful, and empowering communicative physician-patient context is proven to improve patient outcomes, [29, 30] there are real barriers in regards to the enactment of this kind of care because of the inherent power imbalance in the physician-patient dyad characterized by physicians’ possession of expert knowledge.

### The nature of language and meaning

“Language” used in this study is a meaning making system that is always co-constructed and shaped by different gradients of power [31]. The power of language and words is tied to the legitimacy of the words and of the legitimacy of the person who utters them, a belief which words themselves cannot produce but is determined in relationship [32]. With this perspective, we can observe how the language strategies physicians use when interacting with patients is a reflection of their habitus - produced in part from exposure to the field of medicine, a field imbued with symbolic power.

## Methods

### Research design

This current research was part of a larger study that explored how physicians conceptualise their teaching and consultation practices and their thoughts about their professional development in these roles. For this current study we wanted to understand how physicians perceive power relations in the physician-patient encounter. To achieve this, 30 physicians were interviewed and the transcribed data analysed using an inductive thematic approach further described below.

This study was conducted within four Royal College specialties at the University of British Columbia, Canada: Internal Medicine, Surgery, Pediatrics, Psychiatry as well as Family Medicine. These five disciplines were selected as they represent the top five choices of Canadian Medical Graduates and top five available residencies in the CaRMS match [33]. In addition these five disciplines represent disciplines with a mix of both outpatient and inpatient care. While Family Medicine is not a Royal College Specialty, it represents a significant number of providers and trainees and it is conceived using a similar framework as the Royal College of Physicians and Surgeons of Canada [34]. We received ethical approval from the University of British Columbia’s Behavioural Research Ethics Board to conduct the study.

### Population, sample and data collection

We used purposeful sampling to capture a wide range of physicians to ensure we could fully understand the topic under study. The co-investigators on the larger study represented one of the five disciplines, and thus identified colleagues that fit the study’s inclusion criteria of having at least five years of experience in teaching patients and trainees. An email introductory letter that described the larger study was sent to all colleagues identified by the study’s co-investigators. Approximately 4 (70 %) out of 6 (100 %) of those physicians initially contacted in each discipline were interested in participating, and were subsequently sent a consent form prior to the interview taking place. To recruit the additional two participants we used a snowball sampling technique which involved asking those interviewed if they could provide names and email addresses of colleagues who fit our recruitment criteria. We then contacted the new recruits by sending an introductory email describing the study. The interviews were held either in person at the participant’s office (*n* = 12, 40 %) or on the phone (*n* = 18, 60 %) and were approximately 1 to 1.5 h in duration. The majority of interviews were conducted by the first author (LN) and the remaining by a research assistant, all interviews were recorded and transcribed. After the first six interviews were transcribed, the research team (LN & T S-H) met and reviewed the transcripts to fine tune the interview protocol.

### Data analysis

In this exploratory analysis we wanted to focus on physicians’ conceptions of the phenomena of power in the physician-patient encounter, and thus this present analysis drew on a subset of data from the larger study: We explicitly asked two open-ended interview questions within the larger study: “*Do you find that there are power relations in your interactions with patients*?” And “*How do you deal with these power relations if you experience that they are there?”*. and in this current analysis, we focused on responses to these two interview questions. Follow up questions and probing techniques were also used to stimulate more information, such as “*can you tell me more about that?*” or “*right, I see”*. However, we also coded all content within the full transcripts and analyzed any data found that highlighted physicians’ perceptions of power dynamics as they unfold with patients in the clinical context.

Analysis of data began by multiple readings of the verbatim transcripts. We then used LeCompte and Schensul’s [35] approach to analyzing qualitative data that involves a systematic process that takes place in three stages: (1) Item analysis, (2) Pattern analysis, and (3) Structural analysis. We used each of LeCompte and Schensul’s three tiered inductive strategy as this analytic approach involves compiling items together at the specific level and then creating more abstract statements about patterns of relationships in the data to generate overall insights into the topic of interest [35]. Theoretical visibility was also present throughout all stages of analysis to enhance research rigour [36].

**(I)** First, we coded the transcripts for key phrases or tracts of text related to “physician-patient power dynamics”. We used ATLAS.ti qualitative coding software to visually display items in the margins of the program rendering visible the relationship to each other across data sets.

**(II)** We then engaged in pattern analysis, which involved a process of comparison, contrast, and integration and where items are organized, associated with other items, and linked together into higher order patterns. Examples of themes that were generated in this stage were: “awareness of power”; “the contextual nature of power”; and “the strategic handling of power”. These patterns emerged from drawing on prior research studies, the study’s theoretical framework, and our research purpose. For example, in operationalizing the item “awareness of power,” we drew on Bourdieu’s [22] notion that people often experience power differently depending on the different social circumstances or fields they find themselves in.

**(III)** Following pattern analysis, we developed broader themes that involved blending many of the initial codes into finer tuned themes that captured similar conceptual dimensions across the data. These broader themes, for example, were named: “perceptions of holding and managing power”; “perceptions of being disempowered”; and “perceptions of power as non-existent”. These broader themes were then pulled together into a meaningful whole – the interpretation.

We began the interpretation by returning to the original research purpose and reviewing the theoretical and research literature that contextualized the study. This process helped us focus the interpretation on what others can learn from the study and how this is supported by concrete, specific examples.

### Trustworthiness and rigour

We employed strategies of credibility to establish qualitative criteria for trustworthiness and rigour in the research. To ensure credibility in an attempt to compensate for single-researcher bias, LN and T S-H engaged in researcher triangulation by both being involved in the analysis of data [37]. To further enhance credibility and because all steps in qualitative analysis involve acts of interpretation, we also engaged in peer-examination [38] that involved discussing the research process and findings with impartial colleagues. We engaged in an ongoing dialogue with Dr. Glenn Regehr (a well-respected scholar in the field of medical education) and Wendy Hartford (a research assistant who read all of the interview transcripts), comparing insights about our emerging themes and confirming the reliability of our analysis of data. Finally, credibility was ensured by engaging in a thick description of the research process so the reader would be able to follow the research process, such as rational for the study, data collection, and analytic process. These detailed descriptions allow others to be able to determine if the insights can be transferred to their local context and setting [39].

## Results

In the presentation of results, we have selected direct quotes from participants to illustrate the major themes with exactness and precision. To maintain participant confidentiality the quotes are only identified by participants’ discipline (Internal Medicine = IM; Surgery = SUR; Pediatrics = PED; Psychiatry = PSY; Family Medicine = FM. For example, a quote from a family physician would be identified as [FM].

The average length of time physicians had been practicing was 16 years, with the minimum length of time practicing being 6 years and the maximum length of time practicing being 41 years. We found no evidence of physicians’ responses differing by gender or discipline. An even larger data sample using a different research design may have been able to make some supported claims in regards to whether the responses vary by gender, years in practice, and discipline, and if there is a trend in terms of responses predominantly found in one category.

Physicians in this study appeared to be surprised by the interview questions that inquired into their perceptions of power relations in the context of teaching their patients. Many physicians asking us to restate the question or repeating back to us the word power for clarification: “*what…sorry…power*?” [FM]. Once they had a moment to reflect on the questions, physicians in this analysis presented a range of descriptions and interpretations of power relations in the physician-patient encounter. Overall, there were three broad categories of similar responses that highlighted how experienced physicians perceive the nature of power, and the meaning they attribute to power in their role as a healthcare provider to patients. We did not find any evidence of physicians’ responses overlapping into the three different categories. For example, no physicians who were acutely aware of their power (first category) did not in the same interview articulate that power is balanced and dissolved in their equal relationship with patients (third category).

### Category 1: Physicians’ perceptions of holding and managing power

A first category of responses highlighted how physicians perceive themselves to hold power in the context of physician-patient interactions. These physicians reflected on the presence of power extensively, describing how the power dynamic between a physician and patient is explicit and unambiguous: “*Sure, yeah, I think that there definitely are* [power imbalances] *and anyone who says there isn’t would be lying. So certainly, I mean, ultimately with a patient, like the surgeon has the power to make the decision about the treatment and patients come to you and they entrust you with their lives. So-- and it’s amazing to me every day when people will trust their surgeons with-- but really that’s the way society views these things. Where some people would spend much more time and efforts investigating where to get their car fixed than themselves”* [SUR]; “*There is a definite power imbalance which needs to be addressed and modified as much as possible*” [PSY]*;* “*Even though we don’t think of ourselves as being paternalistic and we’ve stepped away from this model there is always a power relationship, in any relationship…acknowledging it and recognizing it is important”* [IM]; “*There is always a power relationship…Patients they have to put their trust in you because you’re talking about and doing things that really they don’t understand or don’t have a background in. So they have to have a faith that you’re doing what’s best for them, and so you have to be cognizant of that to make sure that you never ever take advantage of that role”* [FM]; “*There is a power imbalance.. I mean, you are empowered by the knowledge that you have and the ability to treat patients. So there is an inherent power imbalance…that power imbalance is in knowledge”* [SUR].

This category of physicians believed that because of their medical training and credentials they were in position of power in the physician-patient encounter. This position of power was described as something which needs to be acknowledged and respected by physicians: “*I’ve learned very early on that the relationship between a doctor and a patient is unequal. You are in a position of power as the doctor. As much as you may try to minimize that, the actual reality is that they have you, in general of course, have you on a pedestal. You have to be respectful of that differential nature of the relationship”* [FM]. This group of physicians described how it was their ethical responsibility to be cognizant of the effects of power in their relationship with patients, and to not take advantage of their position of power.

This category of physicians believed that because patients trust their authority and knowledge, they have an inherent responsibility to act in the patient’s best interest by “managing” their position of power with integrity. They spoke at length about the deliberate strategies they use to “handle” power in order to engage in a professional and ethical relationship with patients. We found that these strategies executed through language fell into four different descriptions of power management: (1) the exertion of power (e.g. “pull the power card” [IM] by speaking over interrupting family members or making decisive clinical decisions); (2) the sharing of power (e.g. engaging in collaborative shared decision making practices and imparting medical knowledge); (3) the moderating of power (e.g. “humanizing” [PED] themselves by having the patient call them by first name and disclosing a similar personal experience); and (4) the relinquishing of power (e.g. accepting when the patient does not go ahead with their treatment recommendation or accepting when patients seek and use alternative therapies). For example, a family physician who believed that power was always present in her interactions with patients, described how she shares power through imparting medical knowledge and inviting shared decision making practices: “*One of the benefits of being in family practice is that I think they’ve got an ongoing relationship with you. And I think in the best scenario they know that you’re there for their benefit and their welfare. And so I would hope that, you know, in situations where you’re imparting information for their good that you’re doing that with the best of intentions…I see information as power for them, really. If they’ve got information then they’re-- they’ve got that ability to change things up so I’m able to give them that information*”. When and how experienced physicians in this study chose to strategically handle power was never stable or uniform, but was based on astute interpretations of situational context.

### Category 2: Physicians’ perceptions of power as waning

A second category of responses highlighted how physicians perceive that physician power is waning in the context of physician-patient interactions. Some of the physicians in this group expressed a sense that their power is diminishing in the context of a changing healthcare culture that encourages patient rights, patients as consumers of healthcare, and informed patients. One physician captured this sentiment in the following quote: “*It’s* [power dynamics with patients are] *rare, I think things have shifted. I think you’re seeing that patients probably think they have more power. I think because there’s more consumerism within medicine people have a U.S.-style consumerist way/approach where ‘I have all the information, you should do this treatment because I think this is what I need’. I think that power differential* [exists] versus *I’ve come to you as an expert in this field…I’m not trying to be egotistical. This is what my opinion is. So, I’m seeing actually that power differential…the trickier part* is *when the patient is not agreeing with any care plan that I come up with. So if it becomes a consistent pattern then I often at that point will say, you know, we obviously have a difference in philosophy in terms of how you wish to be treated. Perhaps it’s best that you see either another physician”* [IM].

Overall this group appeared to conceptualize the physician-patient encounter as site of struggle, often describing how tensions emerge when patients make unreasonable demands on the physician. For example, when asked about power dynamics with her patients, a physician described how patients can be unreasonably demanding, and sometimes bullies: “*Yes, there are problems with patients. Mostly people with personality disorders, that may be unfair to say, but people who come in and feel that they know best, come in demanding what they want or…with very specific demands. And so there can be a little bit of a power struggle there…very occasionally they can be a bit bulliesh”* [FM]*.* For those experienced physicians who perceived their power waning in the context of the physician-patient encounter, there was no elaboration on the mechanisms they employed to strategically handle power when interacting with patients.

### Category 3: Physicians’ perceptions of power as non-existent or irrelevant

A third category of responses highlighted how physicians perceive that power dynamics are non-existent or irrelevant in the context of physician-patient interactions. Individuals in this group perceived there to be an absence of power: “*Oh No,* [there are no power dynamics] *unless they* [patients] *have a major personality disorder, that’s rare too, right*” [PSY] or “*I think patients have quite a bit of trust, you know, I don’t find* [power dynamics] *not a power struggle, no, not at all*… *I think my personality’s probably easygoing. It’s hard to get into a power struggle”* [PEDS]. While others in this group perceived power to be dissolved through an equal and balanced power relationship. These physicians describe how they are on a level playing field with patients, which they emphasize has the essence of a collegial and friendly relationship. One physician captured the notion that his interactions with patients were situated in a flat hierarchical power structure in the following quotation: “*A lot of patients really want to be an equal partner in the learning. And some of them are very intelligent and they will ask you difficult questions. And that’s fine, I kind of like that”* [IM] or “*I think the power is more when it’s a doctor-medical trainee interaction…With the patients it’s a little bit different. I hope there’s no power problem coming into my interactions. I’m trying to enable them-- in trying to empower them”* [FM]*.* For physicians in this group, power was not a meaningful or important concept in the context of delivering healthcare to patients, either because they perceived that it does not exist OR because they perceived that power dissolves through a balanced empowering physician-patient power relationship. This group of experienced physicians, who perceived power dynamics as non-existent or irrelevant in their interactions with patients, did not provide any further insights into power as it manifests in the physician-patient encounter.

## Discussion

Physicians’ surprised reaction at our questions related to power dynamics in their encounters with patients suggests that power may not be a concept that physicians routinely reflect upon. Bourdieu’s theoretical concepts of habitus and doxa can be applied to make sense of this lack of reflection of physician power as part a process that develops and (re)structures medical students’ habitus through contact with the broader institutional and organisational culture(s) of medicine [24], a socialization subconscious process that does not traditionally involve discussions or considerations of one’s position of power within the healthcare profession hierarchy. In particular, Bourdieu’s notion of doxa is a useful conceptual tool to make sense of how medical education and training results in the construction of a medical habitus where there are many taken for granted truths that are internalized subconsciously [23]. However, as we engaged more with the data and deepened our analysis, it became clear that this one size fits all application of Bourdieu’s notion of habitus and doxa is incapable of explaining the variance in each three categories of responses in respect to experienced physicians perceptions of power in the physician-patient relationship.

Although trained within the same institutional and organizational culture of medicine, physicians’ varying interpretations of power relations in this study suggest that there is a crucial intersection of agency, context, time, training, and practice which are linked to perceptions of social space and/or the social position an individual possesses. Our findings thus reveal that physicians who all acquire a medical habitus may in fact have a flexible generative [40, 41] medical habitus that adapts to changing circumstances through varying exposure to different social fields within medical training and practice, and further shaped by individual factors, such as values, tastes, beliefs and preferences. These individual values, tastes, beliefs and preferences are socially determined and shaped by experiences as they navigate different social fields within medicine (e.g. the formal curriculum and the hidden curriculum), [42] and the different social fields they are exposed to outside of medicine over time. With this insight, we further triangulated into the analysis Bourdieu’s theoretical concepts of structure and agency to make sense of how the social institution of medicine constrains and enables dispositions, action and perceptions, while accounting for the important interplay of individual action and agency [43]. The coalescing of the many factors that shape physicians medical habitus explains the production of their varying perceptions of power relations in the micro level field of physician-patient interactions, illuminates “the world not as imposing itself immediately and uniformly on all social agents, but as realized through complex processes involving the expectations and hopes of agents themselves…([40](pp.71)).

Following this in depth integration Bourdieu’s social theory to understand the nature of each three categories of responses, we present the implications and/or insights that can be drawn from each group.

For the group of physicians who perceived their power to be waning in their interactions with patients, we believe future research could explore physicians’ language strategies and interpersonal complexities that may arise from physicians’ perceptions that their medical authority is declining in a cultural climate of patient autonomy and empowerment. Furthermore, for the group of physicians who perceived that power dynamics are non-existent or irrelevant when interacting with patients, we question what the implications are for communicative approaches and the patient experience when physicians do not perceive power dynamics to hold any significance in the clinical encounter. We believe these foci, and other related foci, deserve exploration and reflection and are to be important areas of future investigation.

For the group of physicians who were reflective about how they held and managed power, they demonstrated an astute awareness of their power, which suggests a type of reflexive awareness of their internalization of the social and cultural structure of medicine and the way it interacts with the field through practice. This group of physicians appeared to be aware of the legitimized institutional medical power [22] available to them, yet they describe how they do not necessarily “share” this power in any given situation. Physicians in this group ultimately draw our attention to a “handling” of power that is not always as straightforward as “sharing” per se, but is in fact context-specific*.* Rather than being “shared” uniformly, power appears to be moderated by a range of strategies (exerting, sharing, moderating, relinquishing) executed through language to meet physicians’ purposes of cultivating an ethical therapeutic relationship with patients. In other words, such physicians who are aware that they always hold power by virtue of their cultural and symbolic capital [22] are deliberate in how they ethically handle power through language strategies to serve the patient’s best interest. The way this group of physicians use language strategies to handle their power, aligns with Bourdieu’s notion that language is not simply a means of communication but a medium of power [22].

Although this later group illuminates some of the ways experienced physicians are aware of the power available to them, we recognize that power is inherently relational and cannot be “owned” by physicians. As language and meaning are co-constructed and always contextualized by gradients of interpersonal and institutional power, [31] patients play a significant role in the way power plays out in the medical encounter as they must **(1)** recognize the hierarchal position and legitimate authority of physicians who wield power and **(2)** be complicit in physicians’ strategies for handling power [22]. In other words patients must internalize and believe in the rules of the game within this particular field (i.e. illusio), [44] and be complicit in the rules of the game.

### Limitations

The purpose of our study was to gain insights into the different ways experienced physicians perceive power (e.g. how they invoke power and for what purposes) in the clinical encounter. This study that used 30 qualitative data sources was appropriate for developing meaningful themes that resulted in rich insights into the topic of interest. This study needs to be understood as an in depth exploration of physicians’ perceptions of power relations in their interactions with patients in one small sample of participants documented in a particular space and point in time. The thick description and rich insights generated from this particular context offers a starting point for others to extend insights into the ways physicians perceive power in their interactions with patients in a variety of cultural contexts.

### Implications for training

Bourdieu has been charged with being too deterministic in his theoretical approach, [45] yet he does invite possibilities for reflexivity which he describes as “the systematic exploration of the un-thought categories of thought that delimit the thinkable and predetermine the thought”, ([44](pp.40)). Our data suggests it is possible for physicians to be astutely aware of their power and able to handle their power deliberately in reaction to context, which was so vividly described in the first category of responses. We believe that medical education and training and ongoing professional development can play a key a role in raising physicians’ awareness of their position of power and introducing strategies that will enable them to manage and handle their power when practicing medicine. We suggest that early-career and ongoing professional development training should include opportunities to cultivate: **(a)** an awareness and the capacity to be reflexive of physician power and how it plays out in various interactions and **(b)** communication strategies for physicians to “handle” power with insightful deliberation in a range of clinical encounters. Further, we suggest that the depiction of communication in international specialist physician competency frameworks (e.g. Canada’s CanMEDS; the USA’s ACGME; the UK’s GMC), [46–49] that are used for accreditation, evaluation and examination purposes and have such far reaching implications for medical training and practice, broaden to include the contextual and power laden nature of communication and meaning.

### Recommendations for future research

To understand richly the nature of power in the physician-patient encounter, future research could consider both physicians’ and patients’ perceptions of power relations in the clinical encounter. To deepen even further this exploration we might be better positioned to examine how power flows through *all* interpersonal interactions (i.e. family members, partners, friends, other healthcare providers, and so forth) that contextualize the physician-patient encounter - given that the physician-patient micro level “field” is comprised of a myriad of complex interpersonal relations of power that unfold in clinical and community settings.

## Conclusions

One of the central values underpinning patient-centered care is the equal sharing of power that can be enacted through communication practices like shared decision making [15–19]. However, power cannot really be “owned” by physicians, but rather is activated through a relational dance in the therapeutic encounter with patients. Although the “sharing of power” is an overarching goal that we appropriately seek to achieve in modern patient-centered healthcare, our analysis highlights how this concept does not fully capture the complex ways experienced physicians perceive, invoke, and redress power when interacting with patients in the clinical encounter. Physicians’ always have power available to them through their cultural and symbolic capital legitimized by the institution of medicine, and evidently those who are aware of this power strategically share, exert, moderate and relinquish power in response to situational context to best meet the needs of patients. We believe that medical education training that integrates the insights from the group of physicians who were aware of their power and use it strategically can better prepare novice and practicing physicians to enact patient-centered therapeutic communication. We suggest that medical educational initiatives socialize and habituate physicians to be reflective, analytical, and creative, in their “handling” of power in a way that is attune to context in clinical encounters.

### Ethics approval and consent to participate

This study received approval from the University of British Columbia’s Behavioural Research Ethics Board on May 29^th^, 2012. Reference number: H12-00022. All participants consented to participating in this study.

### Consent for publication

Not applicable.

### Availability of data and materials

Due to the sensitive nature of the raw data on which the conclusions of the manuscript rely, it is not publicly available. Please contact the authors for further information.
